# Supplementary material for: Rapid response to hemorrhagic fever emergence in Guinea: community-based systems can enhance engagement and sustainability
Source: PLoS One. 2025 Sep 8;20(9):e0321164. doi: 10.1371/journal.pone.0321164 (PMC12416637; doi:10.1371/journal.pone.0321164)
Supplement: S1 File — (DOCX) [file pone.0321164.s001.docx]

**S1: Thematic guide for Focus Group Discussions**

**Objectives:**

1. Describe and analyze the responses or actions put in place by the community and local and decentralized technical staff following an alert and/or an unusual event
2. Describe and analyze the responses or actions put in place during the 2013-2016 and 2021 Ebola and 2021 Marburg epidemics
3. Identify the needs and constraints encountered by the community following an alert and the implementation of response actions
4. Describe and analyze the expectations of all actors involved in the disease response process

| **Themes** | **Specific objectives** | **Proposed questions for the record** |
| --- | --- | --- |
| Alerts and alarming events | - Identify events that raise alerts within communities - Identify the actors involved - Identify the associated needs - Wait after an alert | 1. What are the events happening here that would alert/concern your community or put it in an alarming situation? 2. How do you identify these events? Mention of a community health alert? 3. What do these alarming alerts or events represent in your local area (impacts)? 4. Which are the most critical times of year or seasons in terms of alarming events? 5. What was the last alarming event the community had to deal with? How did it go? 6. To whom would you report these alerts and events first, second, and so on, and why? 7. What are your needs in the event of an alert (immediate needs to raise an alert, or needs for continuity)? 8. What do you expect when you raise an alert or report signals of alarming or unusual events (what they expect of themselves, of decentralized technical staff, of local elected officials, etc.)? |
| Response actions or measures | - Identify the immediate measures put in place in the event of health and/or unusual events in the community - Identify special measures and/or precautions - Identify the needs, constraints and barriers of the response - Identify the existence of response plans - Know the key areas of collaboration in the response implementation | 1. What immediate response actions do you put in place when you have an alert or unusual case (before technical staff intervene to support communities, or hierarchical authorities to support technical staff)? 2. What do you do, for example, when carcasses of domestic or wild animals are found, or when there is a death in the community or sudden mortalities (provide case examples relevant to the group when asking the question)? 3. Are there any other precautions or special measures you take in relation to these cases? 4. Who is involved in response activities within the community? And how are those activities implemented in the community ? 5. What are your needs during the response activities in the community? 6. What are the constraints and barriers you face when implementing response actions (e.g., when domestic or wild animal carcasses are found, or when there are unexplained community deaths)? 7. Is there a disease response plan in your community, and can you describe it (adapt the question according to the target group)? 8. How do you feel about the way stakeholders collaborate when response actions are implemented (with you, with other actors, with local elected officials)? |
| Stakeholders’ perceptions to response measures (in relation to the Ebola and Marburg viruses or recurrent diseases in the area) | - Identify stakeholder participation in the response implementation - Identify stakeholders’ perceptions with regard to the response measures put in place during previous epidemics - Identify the reasons for resistance to response measures - Identify constraints and levers for engagement | 1. During previous epidemics, were you required to put measures in place to limit the spread of diseases? 2. If so, what measures did you put in place (give an example of a case if possible)? 3. What do you think of these response measures (adapt the question to the group being interviewed)? 4. Which aspects of implementing the measures were easy for or within your community? 5. Which ones were more difficult/restrictive? And why? 6. What obstacles/barriers did you encounter when implementing these measures? 7. What response measures would be most beneficial for your community and why? 8. What measures do you think would be more effective? And how can they be implemented in a sustainable way? 9. Can you tell us why populations sometimes oppose the response measures implemented by the authorities? 10. What specific needs must be met to be able to put comprehensive long-term response measures in place? |
