# Supplementary material for: Rapid response to hemorrhagic fever emergence in Guinea: community-based systems can enhance engagement and sustainability
Source: PLoS One. 2025 Sep 8;20(9):e0321164. doi: 10.1371/journal.pone.0321164 (PMC12416637; doi:10.1371/journal.pone.0321164)
Supplement: S2 File — (DOCX) [file pone.0321164.s002.docx]

**S2: Thematic guide for In-Depth Interviews**

**Specific objectives**

1. Describe and analyze rapid response measures following an alert
2. Understand measures taken by stakeholders for response implementation during previous outbreaks
3. Identify feasible, rapid, and efficient response actions to prevent zoonotic emergence
4. Identify barriers to implementing a coordinated laboratory response
5. Identify all needs to overcome the obstacles to response implementation by including the laboratory aspect

**Stakeholders**

1. Decentralized technical staff in the subprefectures and prefectures
2. Actors from the local viral hemorrhagic fever laboratories in the village of Guéckédou and N’Zérékoré prefectures
3. Actors from the « Virology Research Center (formerly the Donka hemorrahagic fever center), the Institut Pasteur, and the Guinean Infectious Diseases Research and Training Centre (CERFIG)
4. Managers from the national technical services (National Directorate of Veterinary Services – DNSV, Guinean Office of National Parks and Forest Reserves – OGPNRF, National Health Safety Agency – ANSS, One Health Platform)

**Open-ended questions to keep in mind for semi-structured interviews** (answers to questions during interviews may elicit other questions or follow-ups)

1. What immediate actions could you take when you receive an alert from the community or a community worker about a new or abnormal health event?
2. Can you describe the main activities of your organization’s response unit in relation to the response actions implemented?
3. Who handles these response actions?
4. What are the needs for implementing these actions and/or rapid responses to these alerts?
5. What are the constraints and barriers you face when putting response actions in place?
6. How are these response measures perceived by communities and other actors at the higher hierarchical level?
7. What can be done to ensure that responses are accepted and/or tailored to the concerns of the different entities involved in the response?
8. What can you do with community actors (your hierarchical subordinates) so that they raise more alerts and become more involved in the response?
9. How could you jointly manage an alert and response?
10. How do you feel about the way stakeholders collaborate when implementing response actions (between actors within your organization, with other actors, with other institutions)?
