# Supplementary material for: Rapid response to hemorrhagic fever emergence in Guinea: community-based systems can enhance engagement and sustainability
Source: PLoS One. 2025 Sep 8;20(9):e0321164. doi: 10.1371/journal.pone.0321164 (PMC12416637; doi:10.1371/journal.pone.0321164)
Supplement: S3 File — (DOCX) [file pone.0321164.s003.docx]

S3 : Coding tree

| **CODES**  **Themes** | **UNDER CODE 1** | **UNDER CODE 2** | **UNDER CODE 3** | **Elements to be taken into account in subcode 3** |
| --- | --- | --- | --- | --- |
| **Alerts** | 1. **Identification** | Identification criteria | Health events | Diseases under surveillance |
|  |  |  |  | Diseases with a high impact on people and livestock |
|  |  |  | Environmental events | Flooding |
|  |  |  |  | Bushfires |
|  |  |  |  | Winds |
|  |  | How (through what) | Dead livestock, including wildlife mortality |  |
|  |  |  | Case identified by a healer |  |
|  |  |  | Case identified by community workers through picture boxes and experience |  |
|  |  |  | Case identified from consultation by the decentralized officer |  |
|  |  |  | Suspicion due to abnormal event |  |
|  |  |  | Human deaths in the village |  |
|  |  | Who is sounding the alarm | Hunters |  |
|  |  |  | Livestock farmers |  |
|  |  |  | Healers |  |
|  |  |  | Matrons |  |
|  |  |  | Community workers |  |
|  |  |  | Local elected officials (village chiefs, sector chiefs) |  |
|  |  | Who receives | Community | Chief hunter |
|  |  |  |  | Healer |
|  |  |  |  | Community workers and matrons |
|  |  |  |  | Local elected officials (district manager, sectors) |
|  |  |  | Decentralized technical staff | Head of Veterinary Post |
|  |  |  |  | Head of the Health Center |
|  |  |  |  | Head of Environment Department (Forest Divisions) |
|  |  |  |  | Red Cross |
|  | 1. **Alarming events in communities** | Types | Health | Latest health event |
|  |  |  |  | Consequences |
|  |  |  | Environmental |  |
|  |  |  | Other |  |
|  |  | Impacts | Social |  |
|  |  |  | Economic |  |
|  |  |  | Other impacts |  |
|  | 1. **Needs for communicating the alert** | Work tools |  |  |
|  |  | Financial support |  |  |
|  |  | Other needs | Training |  |
|  |  |  | Legitimacy and/or recognition of actors |  |
|  | 1. **Response actions after an alert** | Community | Population |  |
|  |  |  | Community workers |  |
|  |  |  | Local elected officials |  |
|  |  |  | Opinion leaders (religious, customary, youth, women’s leaders) |  |
|  |  | Technical services | Decentralized technical staff |  |
|  |  |  | One Health Platform |  |
|  |  |  | Relevant prefectural services |  |
|  | 1. **Difficulties in producing the alert** | Financial |  |  |
|  |  | Work tools and technical means | Telephone, means of transport |  |
|  |  | Fear of being reprimanded |  |  |
|  |  | Sociocultural context |  |  |
| **Health information feedback flow** | 1. **Process** | Community members to community workers and matrons |  |  |
|  |  | Community members to technical staff |  |  |
|  |  | Within a sector |  |  |
|  |  | Between sectors |  |  |
|  | 1. **Actors involved** | Community | Citizens (healers, livestock and crop farmers, hunters, loggers) |  |
|  |  |  | Community workers and community matrons |  |
|  |  |  | Local elected officials |  |
|  |  | Decentralized technical services |  |  |
|  |  | NGOs and institutions |  |  |
|  | 1. **Means used** | Community | Direct involvement of individuals |  |
|  |  |  | Phone calls |  |
|  |  | Technical staff | Digital support via tablet and Android phones |  |
|  |  |  | Direct involvement |  |
|  |  |  | Standard or toll-free phone calls |  |
|  | 1. **Difficulties and/or constraints** | Financial | No phone credit for calls and connections |  |
|  |  | Work tools and technical means | Lack of communication means (telephones), problem of recharging batteries |  |
| **Epidemic diseases and/or health events mentioned** | 1. **Ebola 2014–2016, referred to as “First Ebola stint” by the actors** | Source |  |  |
|  |  | Number of cases |  |  |
|  |  | Affected category |  |  |
|  |  | Treatment |  |  |
|  |  | Prevention | How |  |
|  |  |  | By whom (actors involved) |  |
|  |  |  | When |  |
|  |  |  | By what means |  |
|  | 1. **Ebola 2021 (“Second Ebola stint”)** | Source |  |  |
|  |  | Number of cases |  |  |
|  |  | Affected category |  |  |
|  |  | Treatment |  |  |
|  |  | Prevention | How |  |
|  |  |  | By whom (actors involved) |  |
|  |  |  | When |  |
|  |  |  | By what means |  |
|  | 1. **Lassa** | Source |  |  |
|  |  | Number of cases |  |  |
|  |  | Affected category |  |  |
|  |  | Treatment |  |  |
|  |  | Prevention | How |  |
|  |  |  | By whom (actors involved) |  |
|  |  |  | When |  |
|  |  |  | By what means |  |
|  | 1. **Marburg** | Source |  |  |
|  |  | Number of cases |  |  |
|  |  | Affected category |  |  |
|  |  | Treatment |  |  |
|  |  | Prevention | How |  |
|  |  |  | By whom (actors involved) |  |
|  |  |  | When |  |
|  |  |  | By what means |  |
|  | 1. **Other diseases (measles, rabies, PPR, pig diarrhea, blackleg)** | Source |  |  |
|  |  | Number of cases |  |  |
|  |  | Affected category |  |  |
|  |  | Treatment |  |  |
|  |  | Prevention | How |  |
|  |  |  | By whom (actors involved) |  |
|  |  |  | When |  |
|  |  |  | By what means |  |
| **Acts of response and/or response** | 1. **Acts of response implemented** | Community | Citizens, community workers and matrons, local elected officials |  |
|  |  | Technical staff | Local (subprefectural level) |  |
|  |  |  | Decentralized (prefectural level) |  |
|  |  |  | National services |  |
|  | 1. **Actors involved** | Community |  |  |
|  |  | Technical staff | Local (subprefectural level) |  |
|  |  |  | Decentralized (prefectural level) |  |
|  |  |  | National services |  |
|  |  | NGOs and institutions |  |  |
|  | 1. **Roles of the actors involved** |  |  |  |
|  | 1. **Needs** |  |  |  |
|  | 1. **Constraints** |  |  |  |
| **Response flowchart** | 1. **Official organizational chart** | Management of the suspected case | Threshold, response actions implemented, deadline |  |
|  |  | Management of the confirmed case | Threshold, response actions implemented, deadline |  |
|  |  | Unconfirmed case |  |  |
|  |  | Actors involved in management measures |  |  |
|  |  | Means of implementation |  |  |
|  | 1. **Organizational chart as perceived by actors** | Management of the suspected case | How, by whom, whenby what means? |  |
|  |  | Management of the confirmed case | How, by whom, when by what means? |  |
|  |  | Response actions | Community level |  |
|  |  |  | Technical staff level |  |
|  |  | Means of implementation |  |  |
|  |  | Needs |  |  |
|  |  | Difficulties |  |  |
| **Actors’ perception of response and/or response measures** | **Positive and negative perceptions** | Communities’ expectations of technical staff |  |  |
|  |  | Measures deemed beneficial | Why |  |
|  |  | Measures deemed burdensome or difficult to implement | Why |  |
|  |  | Measures considered easy to implement | Why |  |
|  |  | What is needed for the acceptability of the measures | Who is involved? How? What needs must be met to take action? |  |
| **Needs and obstacles for alert and response** | **Needs** | Financial | Per diems, salaries, phone credit, etc. |  |
|  |  | Materials and/or work tools | Protection kits, picture boxes, boots, phones, means of transport, etc. |  |
|  |  | Training | Recognition of diseases, roles and responsibilities of actors, revitalization, risk and means of prevention |  |
|  | **Obstacles** | Organizational |  |  |
|  |  | Surveillance capabilities |  |  |
|  |  | Formalization of roles and responsibilities |  |  |
