# Supplementary material for: Rapid response to hemorrhagic fever emergence in Guinea: community-based systems can enhance engagement and sustainability
Source: PLoS One. 2025 Sep 8;20(9):e0321164. doi: 10.1371/journal.pone.0321164 (PMC12416637; doi:10.1371/journal.pone.0321164)
Supplement: S4 File — (DOCX) [file pone.0321164.s004.docx]

# S4: Description of stakeholders, organizations, and roles

In the context of emerging and re-emerging zoonotic diseases in Guinea, several stakeholders and bodies at three different levels interact to carry out disease surveillance:

1. **At national level:** these employees are referred to in this study as **“national staff.”** They include:

- The Ministry of Health and Public Hygiene, with the National Health Security Agency (ANSS) as the body responsible for disease surveillance;
- The Ministry of Agriculture and Livestock: National Directorate of Veterinary Services, with the National Network for Animal Disease Surveillance (REMAGUI) as the epidemiological surveillance body;
- The Ministry of the Environment, Water, and Forests, with the Guinean Office of National Parks and Nature Reserves (OGPNRF) as the body responsible for managing and monitoring diseases and events in wildlife and reserves;
- The National One Health Committee, with members from various ministries;
- Laboratories such as the Virology Research Center (formerly the Donka hemorrhagic fever center), Guinean Centre for Training and Research in Infectious Diseases (CERFIG), and the Institut Pasteur in Guinea.

These bodies are responsible for implementing the national policy on epidemiological surveillance and diagnosis of priority diseases (e.g., rabies, anthrax, brucellosis, avian influenza, Ebola virus disease, Lassa fever, Marburg virus disease, yellow fever), working in accordance with the national health system and according to the “One Health” concept. These bodies also handle other diseases specific to each sector through their own entities, including human health centers and stations, Epidemiological Treatment Centers (known as CTEpi), veterinary stations, environmental stations and forestry stations spread throughout the country.

1. **At the decentralized level**, these are:

- Regional Departments of Human Health, Livestock and Environment;
- Prefectural Health Department: Director, doctor in charge of the disease (MCM), DHSI_2_ notification staff in DHIS2 (https://dhis2.org/), staff in charge of epidemiological surveillance, Prefectural Epidemic Alert and Response Team (EPARE);
- Prefectural Livestock Department: Director, Animal Health and Surveillance Officer, Animal Production Officer, Mobile Animal Health Unit Officers, Event Mobile Application EMA-i Officer;
- Prefectural Department of Environment, Water, and Forests (Director, Assistant Directors, Parks and Nature Reserves Officer).

Officers at this intermediate level are responsible for implementing national policy on disease surveillance, participating in the development of surveillance manuals and procedures, conducting zoonoses field investigations, preparing monthly and periodic epidemiological surveillance reports, and carrying out other activities related to their various departments. In this study, they are referred to as **“regional or prefectural technical staff”** in the human health, livestock, and environment sectors.

1. **At local level:**
   1. **Decentralized technical staff at subprefectural level**:

- Human health: Head of the health center at the subprefecture capital, heads of health posts in the districts, staff involved in the expanded immunization program, prenatal consultation staff;
- Livestock: Head of the veterinary post;
- Environment: Head of the environment post, also known as head of the forestry unit post.

In this study, they are referred to as **“local staff”** for the human health, livestock, and environment sectors. They are not only members of the One Health subprefectural platform, but are also responsible for handling epidemiological disease surveillance, implementing local response alert plans under the aegis of the National Health Safety Agency (ANSS) and the prefectoral supervisory authorities, treating common human and domestic animal diseases, and dealing with forestry issues (logging, bushfires, poaching, etc.).

- 1. Community liaisons or community health, livestock, and environment staff, referred to in the study as **“community workers.”**

These are citizens appointed by local elected representatives on a voluntary basis who can relay awareness-raising messages and information to other populations on specific human health, livestock, and environment issues related to disease recognition, prevention, and control. They are also involved in disease surveillance systems, as they are in closer proximity to the local population and play a key role in alerting and informing people of unusual events and the presence of disease symptoms in the villages.

- 1. Local elected representatives, matrons, opinion leaders, healers, hunters, farmers, herders, woodcutters, teachers, and village announcers were all included in this study and are referred to as **“community members.”**

A community is considered to be a group of people or individuals living in a well-defined area at a given time, sharing common interests and socio-cultural values.

Given that the community is at the interface between humans and animals (including wild animals), it is the first to suffer the consequences of health events (diseases) or other unusual events, but also to benefit from response measures. Community members sometimes participate in alerts (hunters, loggers, village matrons) and in raising public awareness (role played by local elected officials, opinion leaders, and religious leaders, for example).
